# Exercise and/or Cold Exposure Alters the Gene Expression Profile in the Fat Body and Changes the Heart Function in *Drosophila*

**DOI:** 10.3389/fendo.2022.790414

**Published:** 2022-03-28

**Authors:** Ting Huang, Xiaoyi Jian, Jinglin Liu, Lan Zheng, Fang Qiu Li, Ding Meng, Tongquan Wang, Shihu Zhang, Yi Liu, Zhilong Guan, Jiadong Feng

**Affiliations:** Key Laboratory of Physical Fitness and Exercise Rehabilitation of Hunan Province, Hunan Normal University, Changsha, China

**Keywords:** exercise, cold expose, cardia function, ucp4c, SIRT1

## Abstract

The major reason of human morbidity and mortality is obesity and related diseases. Brown adipose tissue (BAT) is associated with low total adipose tissue content and a lower risk of type 2 diabetes mellitus. Studies have shown that exercise and cold expose may induce browning. In this study, we verified (1) whether exercise and/or cold exposure can improve the expression level of *ucp4c, serca, ampkα, camkII, sirt1, octβ3r*, and *hamlet*; (2) if these interventions can save cardiac dysfunction induced by a high-fat diet (HFD) in *Drosophila.* w1118 (wild-type) virgin female flies collected within 8 h after eclosion were divided into eight groups: the normal feed control group (NFD-C), the normal feed exercise group (NFD-E), the normal feed cold exposure group (NFD-CA), the normal feed exercise/cold exposure group (NFD-EC), the HFD control group (HFD-C), the HFD exercise group (HFD-E), the HFD cold exposure group (HFD-CA), and the HFD exercise/cold exposure group (HFD-EC). After exercise and/or cold exposure for 7 days, the mRNA expression levels of *ucp4c, serca, ampkα, camk II, sirt1, octβ3r*, and *hamlet* were tested by qRT-PCR, and m-mode was used to assess cardiac function. In addition, we assessed the triacylglycerol (TAG) levels, motor ability, fat mass (by Oil Red O [ORO] staining), and morphological features. The results of TAG, ORO staining, and morphological features all indicate that after interventions, body size of *Drosophila* was smaller compared with the control group, irrespective of the feeding patterns. The mRNA expression levels of *ucp4c, serca, octβ3r, hamlet, ampkα, camkII, and sirt1* were changed to varying degrees under different intervention states (exercise and/or cold exposure). Cold exposure and exercise/cold exposure partly improved cardiac function and the normal fruit flies’ cardiac function and exercise ability. However, after exercise intervention, exercise ability and heart function were improved in both HFD and normal-fat diet (NFD) fruit flies. In conclusion, different intervention states (exercise and/or cold exposure) can change the mRNA expression levels of *ucp4c, serca, octβ3r, hamlet, ampkα, camkII, and sirt1*. Exercise is the most effective way to restore HFD-induced cardiac dysfunction.

## Introduction

Obesity increases the risk of metabolic diseases and cardiovascular disease and impairs exercise capacity (1). Brown adipose tissue (BAT) is emerging as a promising and interesting target for therapeutic intervention in metabolic disease and obesity. A large number of studies have shown that BAT thermogenesis is efficiently activated upon repeated cold exposure or exercise and is important in the regulation of body weight and energy expenditure (2–4).

UCP1-mediated mitochondrial thermogenesis is a hallmark of both brown and beige adipocytes and is commonly used as a molecular marker to identify brown and beige adipocytes (5–7). For beige fat thermogenesis, SERCA2b is required. SERCA2b is important in both the presence and absence of UCP1. When UCP1 is absent, beige fat enhances tricarboxylic acid metabolism, glycolysis, and pyruvate dehydrogenase activity to dynamically expend glucose for ATP-dependent thermogenesis by the SERCA2b pathway (8). Moreover, the PRDM16 and β3-adrenergic receptors, as beige markers, also play an indispensable role in browning (9, 10). Exercise and cold exposure are two factors that induce browning, and during the process, the genes that control energy expenditure also play an integral role. A number of studies have mentioned that AMPK, CAMKII, and SIRT1 play a potentially important role in regulating the thermogenic program that allows for brown and beige adipocytes to take up more glucose and burn more lipid through non-shivering thermogenesis (11, 12).

*Drosophila* and human genomes have shown that ~80% of human diseases in which the disease-related gene has been identified have an orthologue in *Drosophila* according to the analyses from Homophila database (13). Moreover, because of its relatively short lifespan, low economic cost, and easy to be fed, *Drosophila* is considered an excellent model for studying cardiovascular disease, metabolism, obesity, and exercise (14–18). However, little research has reported the relationship of cold exposure/exercise, obesity, and cardiovascular disease in *Drosophila*. In the present study, we explored the effects of expression levels of *ucp4 c* (the homologous gene of ucp1), *serca* (the homologous gene of serca2b), *octβ3r* (the homologous gene of β3-adrenergic receptor), *hamlet* (the homologous gene of prdm16), *ampkα* (the homologous gene of ampk), *camk II* (the homologous gene of camk), and *sirt1* (the homologous gene of sirt1) genes in *Drosophila* induced by exercise and/or cold exposure on exercise capacity and cardiac function. This is of great significance for the study of cardiovascular diseases caused by a high-fat diet.

## Results

### Exercise and/or Cold Exposure Reduced the Amount of Fat in Fruit Flies

**Figure 1:**
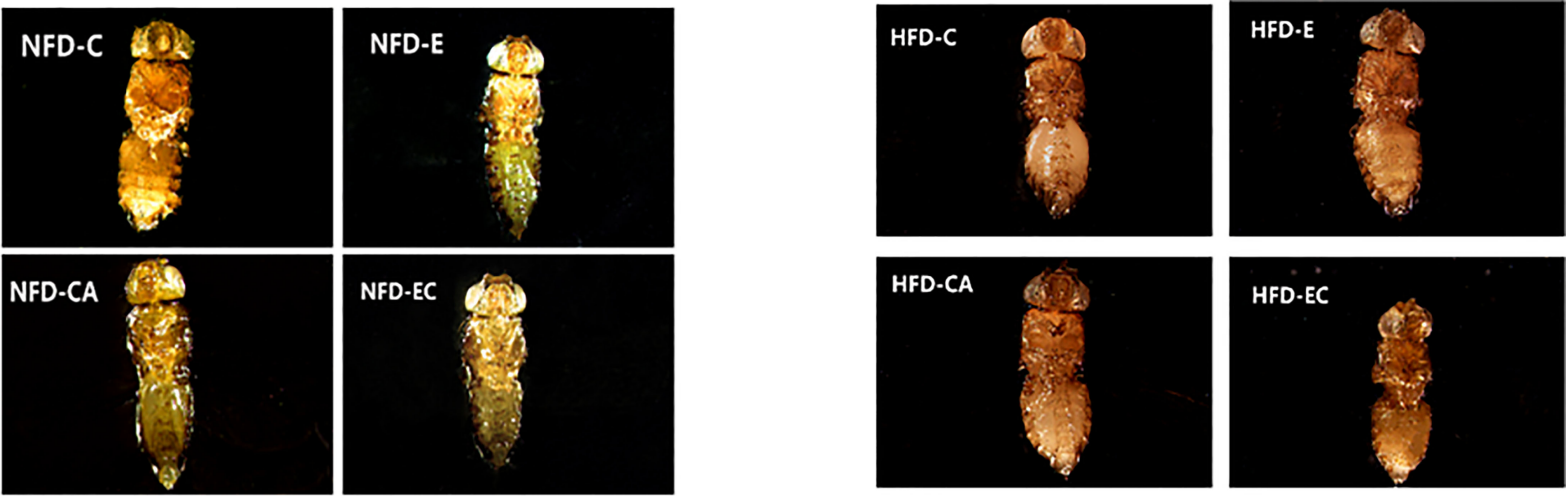
*Drosophila* morphological characteristics. The wings and legs of fruit flies were removed for observation.

**Figure 2:**
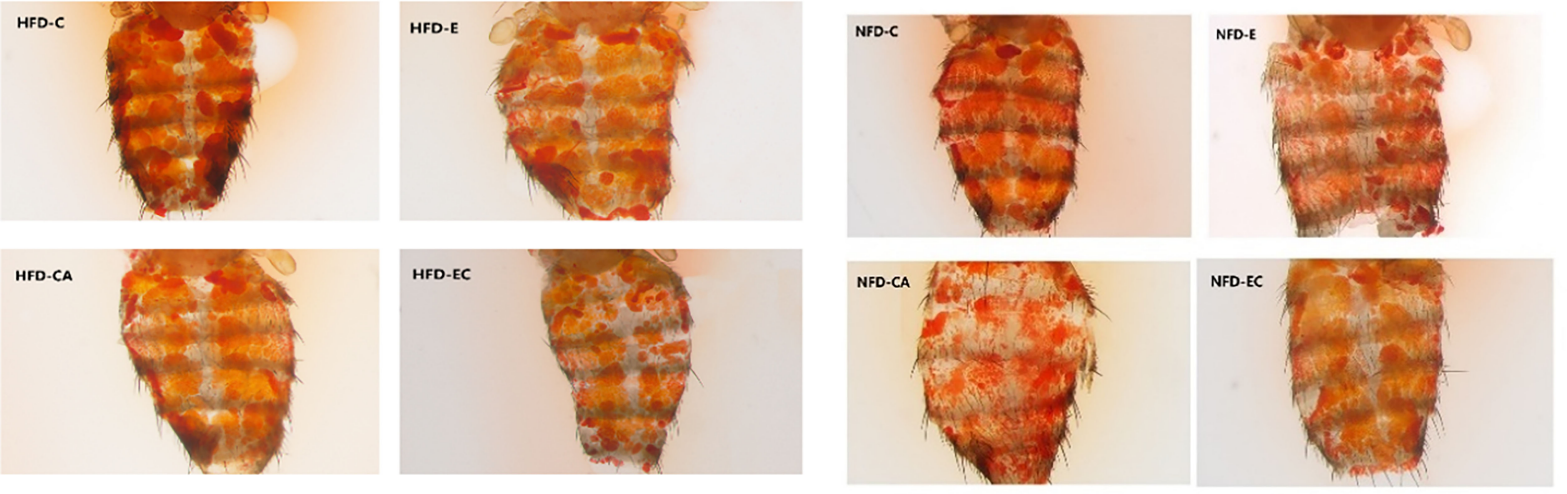
*Drosophila* abdominal ORO staining.

**Figure 3:**
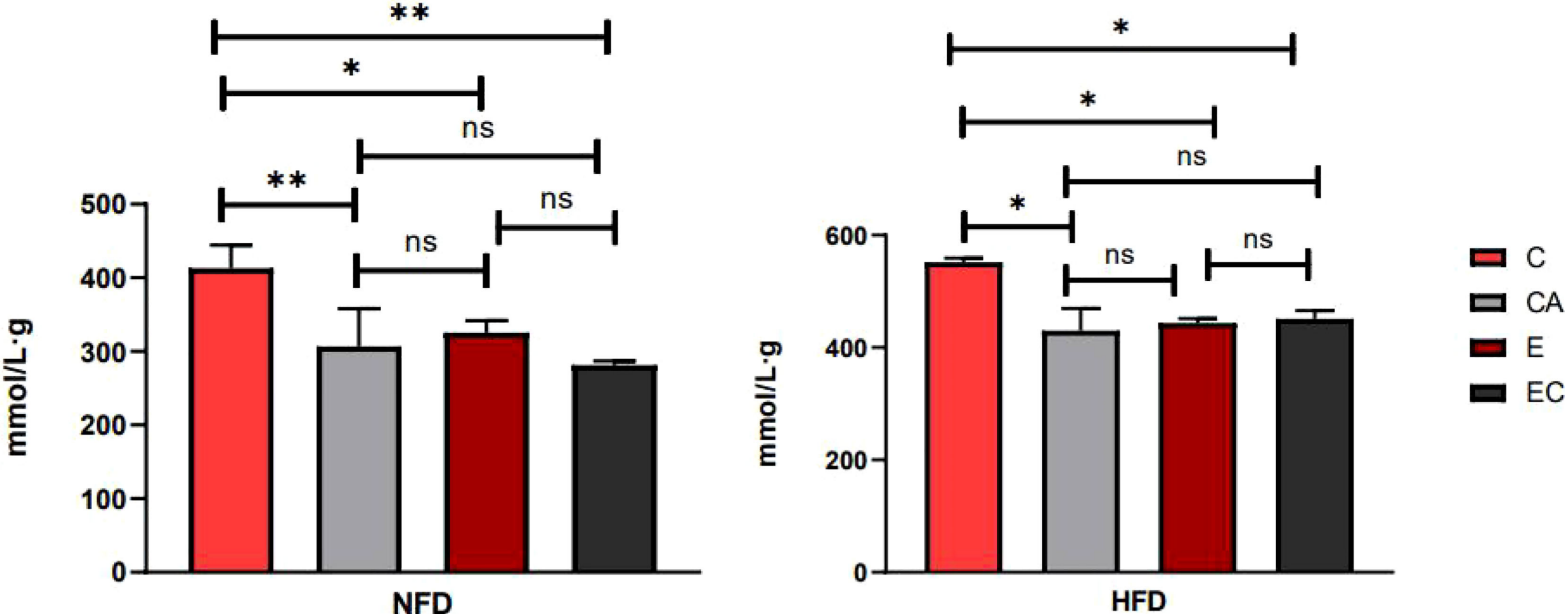
Whole-body triglyceride metabolism in *Drosophila*. *P < 0.05, **P < 0.01, ns P > 0.05.

Triacylglycerol (TAG) is the main lipid storage form in both humans and flies; therefore, quantification of TAG content has always been used to define obesity in flies. According to the multi-factor analysis of variance (ANOVA) of triacylglycerol (TAG) levels (**Table 1**), feeding patterns and interventions exert major effects on TAG levels and have no interaction. In the present study, fruit flies’ morphological characteristics were imaged using a Leica stereomicroscope. As shown in **Figure 1**, high-fat diet (HFD) fruit flies have a larger body size, proving that HFD can induce an obese *Drosophila* model. Moreover, fruit flies had a smaller body size after exercise and/or cold exposure under two different feeding regimes. In addition, as shown in **Figure 2**, the results of our Oil Red O (ORO) staining assay revealed that fruit flies in the control group without exercise and/or cold exposure possessed more adipose tissue. Similarly, as shown in **Figure 3**, in both HFD and normal-fat diet (NFD) *Drosophila*, exercise and/or cold exposure reduced whole-body triacylglycerol (TAG) levels. In summary, all data with respect to the flies’ morphological characteristics, TAG levels, or ORO staining indicate that exercise and/or cold exposure decreased adipose tissue levels.

**Table 1:** Multi-factor analysis of variance of TAG levels.

Parameters ofcardiac function	Source	Type III sum of squares	df	Mean square	F	Sig.
Heart rate	Feeding patterns	1.078	1	1.078	5.164	0.025
	Intervention methods	6.485	3	2.162	10.357	0.000
	Feeding patterns × intervention methods	2.570	3	0.857	4.104	0.008
Hear period	Feeding patterns	0.023	1	0.023	0.502	0.480
	Intervention methods	1.282	3	0.427	9.135	0.000
	Feeding patterns × intervention methods	0.492	3	0.164	3.505	0.017
Diastolic intervals	Feeding patterns	0.132	1	0.132	3.625	0.059
	Intervention methods	0.597	3	0.199	5.470	0.001
	Feeding patterns × intervention methods	0.378	3	0.126	3.463	0.018
Systolic intervals	Feeding patterns	0.011	1	0.011	4.966	0.027
	Intervention methods	0.059	3	0.020	9.074	0.000
	Feeding patterns × intervention methods	0.004	3	0.001	0.664	0.576
Arrhythmia	Feeding patterns	0.270	1	0.270	120.211	0.000
	Intervention methods	0.120	3	0.040	17.756	0.000
	Feeding patterns × intervention methods	0.028	3	0.009	4.110	0.008
Diastolic diameter	Feeding patterns	7186.813	1	7186.813	2.862	0.093
	Intervention methods	77704.678	3	25901.559	10.313	0.000
	Feeding patterns × intervention methods	29669.679	3	9889.893	3.938	0.010
Systolic diameter	Feeding patterns	23478.422	1	23478.422	13.795	0.000
	Intervention methods	17265.249	3	5755.083	3.382	0.020
	Feeding patterns × intervention methods	24360.895	3	8120.298	4.771	0.003
Fractional shortening	Feeding patterns	0.936	1	0.936	198.541	0.000
	Intervention methods	0.118	3	0.039	8.313	0.000
	Feeding patterns × intervention methods	0.017	3	0.006	1.203	0.311
Fibrillations	Feeding patterns	1.247	1	1.247	13.584	0.000
	Intervention methods	3.159	3	1.053	11.475	0.000
	Feeding patterns × intervention methods	0.643	3	0.214	2.335	0.076

### Effects of Exercise and/or Cold Exposure on the mRNA Expression Levels of *ucp4c, serca, ampkα, camk II, hamlet, octβ3r,* and *sirt1*

**Figure 4:**
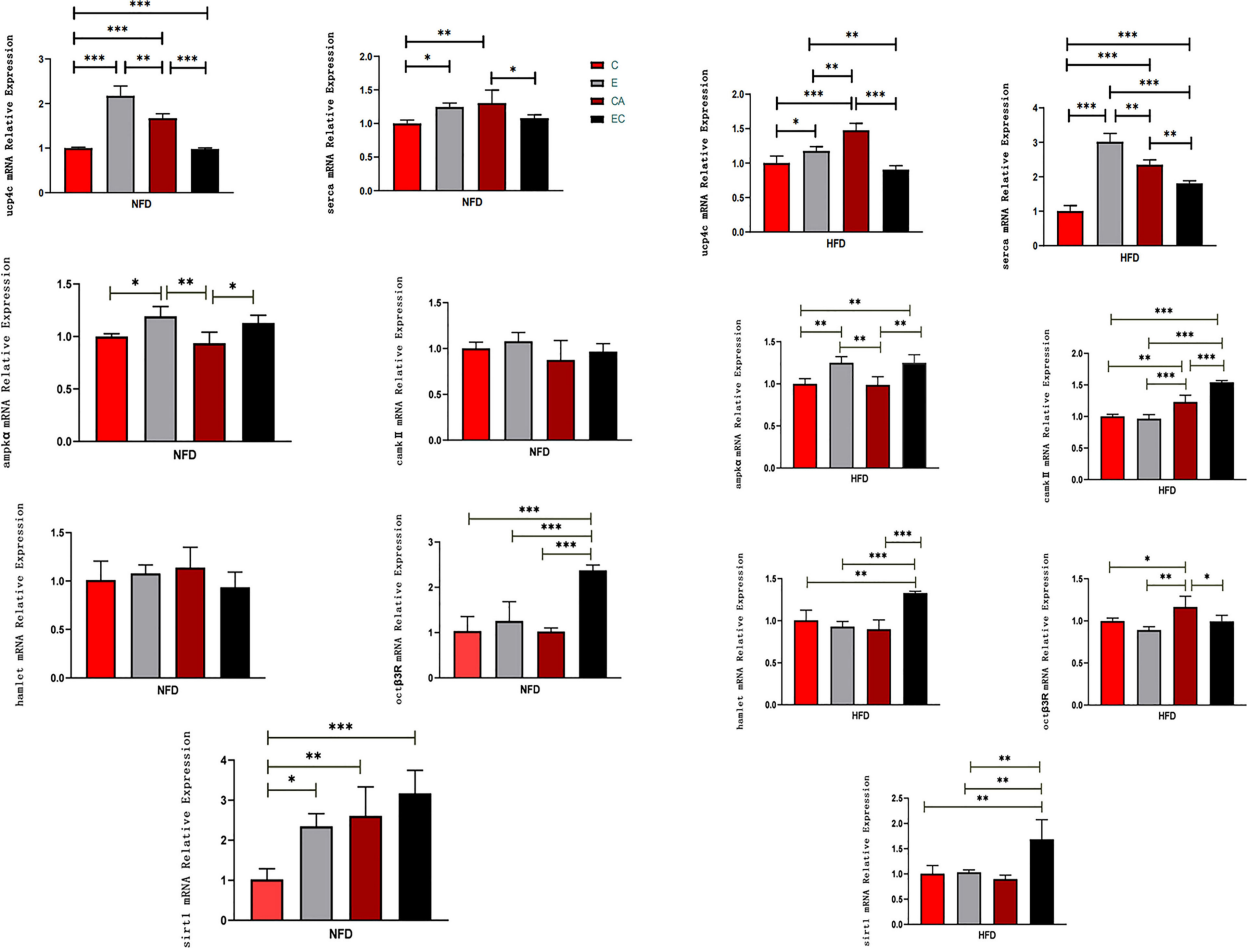
mRNA expression levels of *ucp4c*, *serca, octβ3r, hamlet, ampkα, camk II*, and *sirt1* under different interventions. *P < 0.05, **P < 0.01, ***P < 0.001.

As shown in **Figure 4**, in NFD fruit flies, (1) after exercise, the relative mRNA expression levels of *ucp4c, serca, ampkα*, and *sirt1* were significantly higher, while *camk II, hamlet*, and octβ3r showed no significant change; (2) after cold exposure, the relative mRNA expression levels of *ucp4c, serca*, and *sirt1* were significantly higher, while *ampkα*, *camk II, hamlet*, and *octβ3r* showed no significant change; (3) after exercise and cold exposure, the relative mRNA levels of *ucp4c, octβ3r*, and *sirt1* were significantly higher, while *serca, ampkα, camk II*, and *hamlet* showed no significant change. In HFD fruit flies, (1) after exercise, the relative mRNA expression levels of *ucp4c, serca, and ampkα* were significantly higher, while *camk II, hamlet, octβ3r*, and *sirt1* showed no significant change; (2) after cold exposure, the relative mRNA expression levels of *ucp4c, serca, camk II*, and *octβ3r* were significantly higher, while *ampkα, hamlet, and sirt1* showed no significant change; (3) after exercise and cold exposure, the relative mRNA levels of *serca, ampkα, camk II, hamlet*, and *sirt1*were significantly higher, while *ucp4c* and *octβ3r* showed no significant change.

### Effects of Exercise and/or Cold Exposure on Locomotor Capacity in Different Feeding Conditions

**Figure 5:**
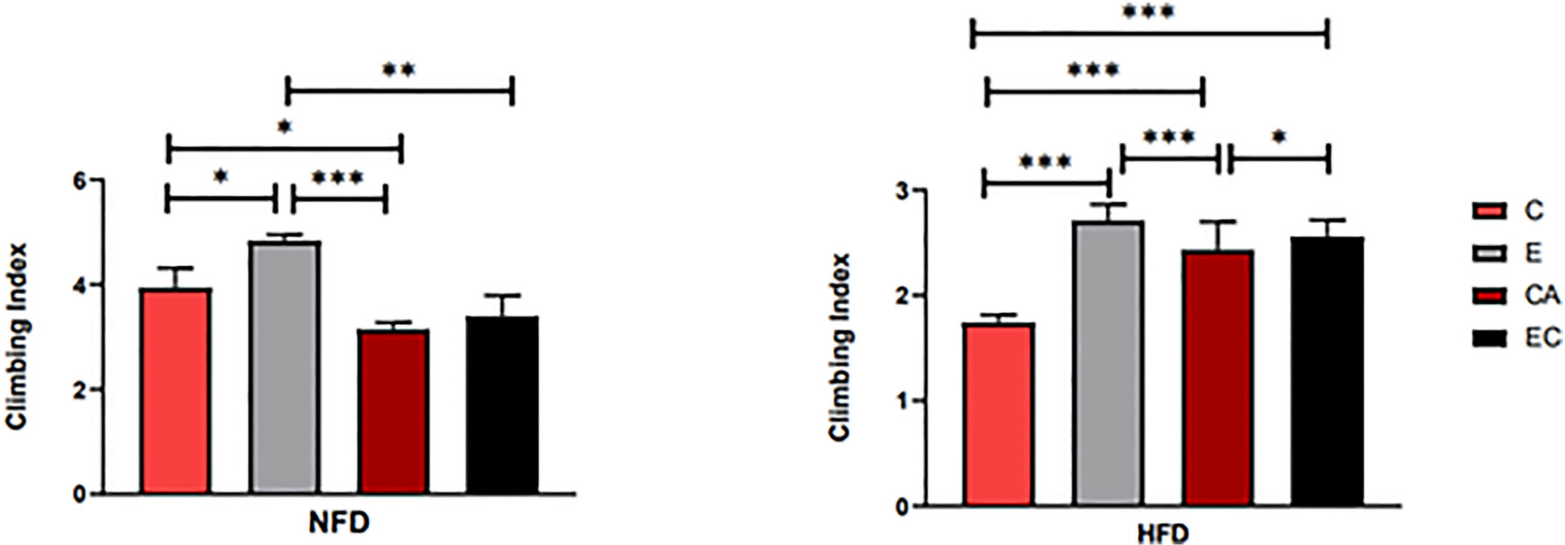
Flies’ locomotor ability under different interventions. *P < 0.05, **P < 0.01, ***P < 0.001.

The climbing index of fruit flies was tested using a climbing device that measures the negative geotaxis behavior (RING) of flies (19). As shown in **Table 2**, feeding patterns and intervention methods have strong effects on locomotor capacity and have interactive effects. According to **Figure 5**, in both HFD and NFD fruit flies, exercise had a positive effect on the climbing index. Interestingly, after cold exposure for 7 days, in HFD fruit flies, the climbing index was significantly increased, while in NFD fruit flies, the climbing index was significantly decreased. As regards fruit flies under exercise/cold exposure conditions, in NFD fruit flies, no significant change in climbing index was observed, whereas in HFD fruit flies, the climbing index was significantly increased (**Figure 5**).

**Table 2:** Multi-factor analysis of variance of cardiac function.

Parameters of cardiac function	Source	Type III sum of squares	df	Mean square	F	Sig.
Heart rate	Feeding patterns	1.078	1	1.078	5.164	0.025
	Intervention methods	6.485	3	2.162	10.357	0.000
	Feeding patterns × intervention methods	2.570	3	0.857	4.104	0.008
Heart period	Feeding patterns	0.023	1	0.023	0.502	0.480
	Intervention methods	1.282	3	0.427	9.135	0.000
	Feeding patterns × intervention methods	0.492	3	0.164	3.505	0.017
Diastolic intervals	Feeding patterns	0.132	1	0.132	3.625	0.059
	Intervention methods	0.597	3	0.199	5.470	0.001
	Feeding patterns × intervention methods	0.378	3	0.126	3.463	0.018
Systolic intervals	Feeding patterns	0.011	1	0.011	4.966	0.027
	Intervention methods	0.059	3	0.020	9.074	0.000
	Feeding patterns × intervention methods	0.004	3	0.001	0.664	0.576
Arrhythmia	Feeding patterns	0.270	1	0.270	120.211	0.000
	Intervention methods	0.120	3	0.040	17.756	0.000
	Feeding patterns × intervention methods	0.028	3	0.009	4.110	0.008
Diastolic diameter	Feeding patterns	7186.813	1	7186.813	2.862	0.093
	Intervention methods	77704.678	3	25901.559	10.313	0.000
	Feeding patterns × intervention methods	29669.679	3	9889.893	3.938	0.010
Systolic diameter	Feeding patterns	23478.422	1	23478.422	13.795	0.000
	Intervention methods	17265.249	3	5755.083	3.382	0.020
	Feeding patterns × intervention methods	24360.895	3	8120.298	4.771	0.003
Fractional shortening	Feeding patterns	0.936	1	0.936	198.541	0.000
	Intervention methods	0.118	3	0.039	8.313	0.000
	Feeding patterns × intervention methods	0.017	3	0.006	1.203	0.311
Fibrillations	Feeding patterns	1.247	1	1.247	13.584	0.000
	Intervention methods	3.159	3	1.053	11.475	0.000
	Feeding patterns × intervention methods	0.643	3	0.214	2.335	0.076

### Effects of Exercise and/or Cold Exposure on Cardiac Function

**Figure 6:**
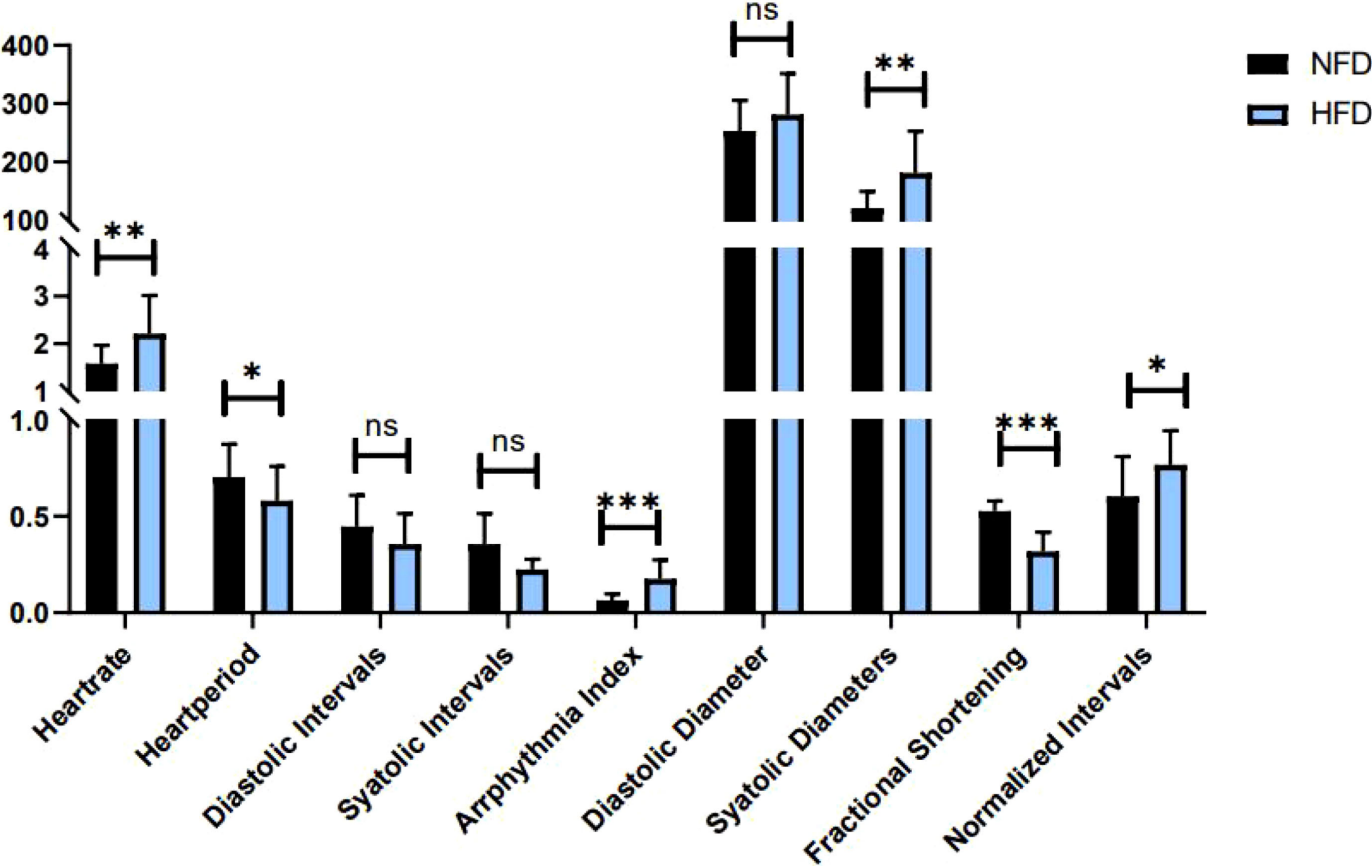
Heart function in HFD and NFD fruit flies. *P < 0.05, **P < 0.01, ***P < 0.001, ns P > 0.05.

Indicators to evaluate cardiac function are abundant. In *Drosophila*, M-mode is a common method. **Table 3** shows the main effect and interaction of cardiac function indices. As shown in **Figure 6**, in HFD fruit flies, the levels of HR, AI, and FL were significantly increased while FS was significantly decreased compared with NFD fruit flies, confirming that HFD reduces heart function. (1) After exercise intervention, in the NFD group, HR, AI, and FL were significantly decreased, while DI, SD, and FS were significantly increased and HP, SI, and DD were not significantly different. In the HFD group, HR, AI, and FS also decreased, and HP, DI, DD, and FS significantly increased. (2) After cold exposure, in the NFD group, HR significantly decreased, and HP, SI, DI, DD, SD, and FS significantly increased. In the HFD group, HR significantly decreased, HP, SI, DD, FS, and FL significantly increased, and others had no significant changes. (3) After exercise/cold exposure, in NFD fruit flies, DD and SD significantly increased, and others had no significant change. In the HFD group, HR significantly decreased and FS and FL significantly decreased.

**Table 3:** Multi-factor analysis of variance of locomotor capacity.

Source	Type III sum of squares	df	Mean square	F	Sig.
Feeding patterns	75692.644	1	75692.644	104.789	0.000
Intervention methods	36609.452	3	12203.151	16.894	0.001
Feeding patterns × intervention methods	1608.249	3	536.083	0.742	0.556

**Figure 7:**
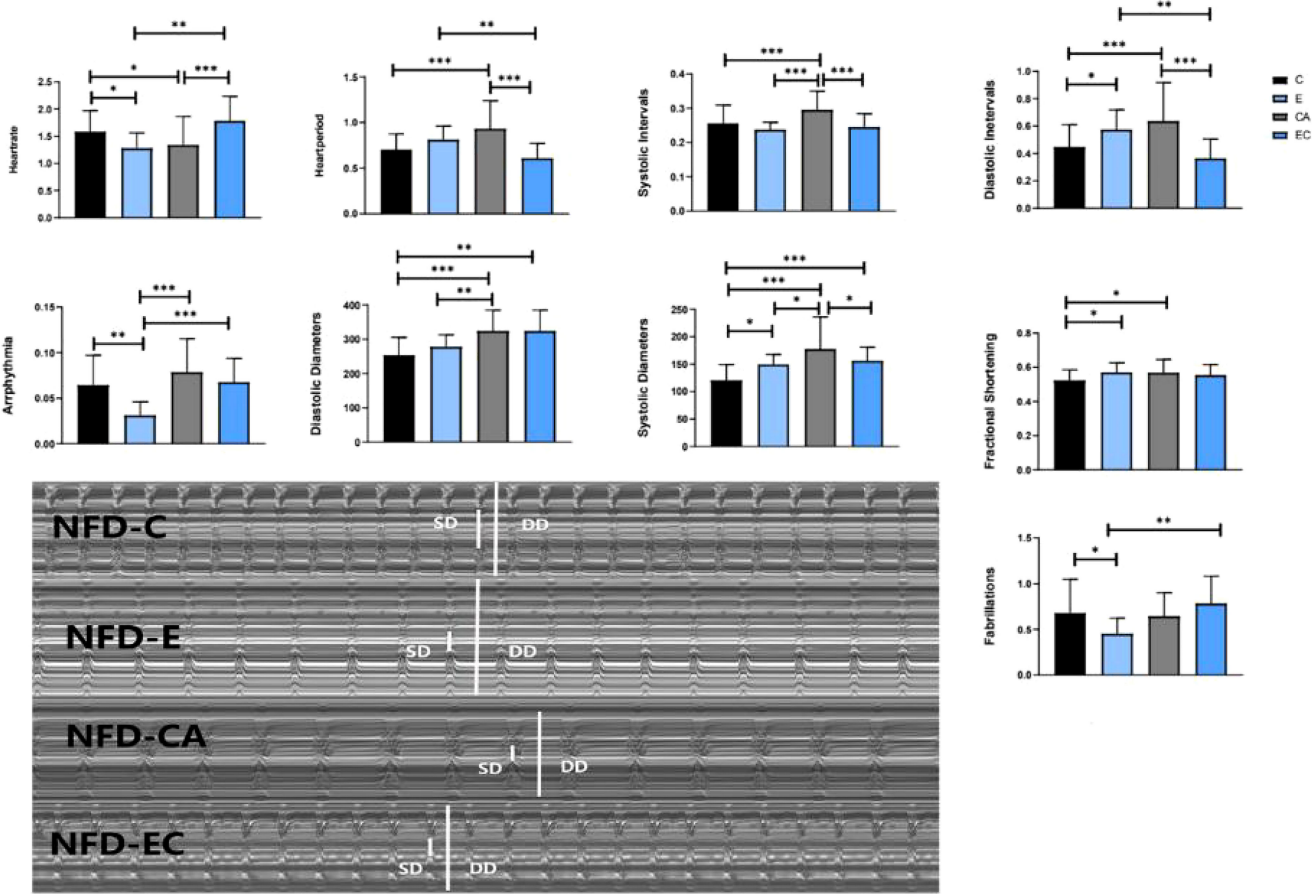
Changes in heart function of NFD fruit flies. *P < 0.05, **P < 0.01, ***P < 0.001.

**Figure 8:**
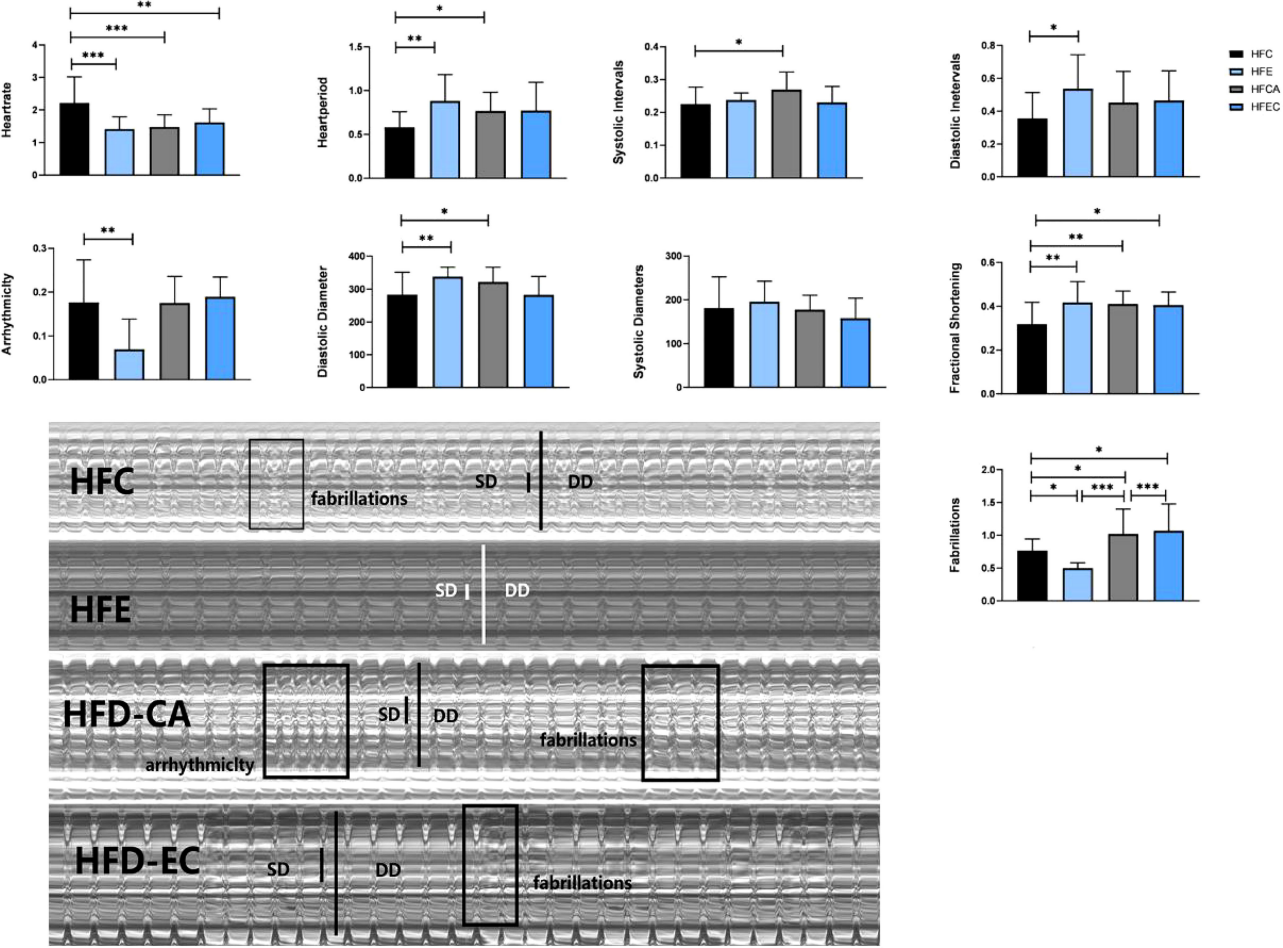
Changes in heart function of HFD fruit flies. *P < 0.05, **P < 0.01, ***P < 0.001.

Comparing these results, we see that HR was significantly decreased irrespective of the intervention, which means that all interventions can decelerate the heartbeat of fruit flies. Moreover, the results shown in **Figures 7**, **8** indicate that regular exercise can restore HFD-induced reductions in cardiac function, strengthen the heart pumping function, and reduce the incidence of AI and FL. However, among fruit flies in the cold exposure and exercise/cold exposure groups, different results were observed between the NFD and HFD groups. In HFD fruit flies, cold exposure or exercise/cold exposure seems not a good way to save heart function as AI and FL significantly increased, which means the risk of cardiovascular disease increased. In NFD fruit flies, after cold exposure or exercise/cold exposure, AI and FL were not significantly changed, but FS was significantly increased after cold intervention, which indicates that cold exposure strengthens the heart pumping function.

## Discussion

Adipose tissue is composed of a complex network that participates in the regulation of different biological functions, such as vascular tone control, glucose and lipid metabolism, and body weight homeostasis. Imbalance of these adaptive homeostatic mechanisms leads to adipose tissue dysfunctionality, which will cause obesity and metabolic disease-associated inflammation or metaflammation (1). For example, adipose tissue expansion, adipocyte hyperplasia, and hypertrophy (increase in cell number and cell size) always lead to obesity (20), which represents a major risk factor for the development of several of the most common medical conditions, such as type 2 diabetes mellitus, dyslipidemia, non-alcoholic fatty liver, and cardiovascular disease. Recently, BAT has emerged as a promising and interesting target for therapeutic intervention in metabolic disease and obesity. UCP1, SERCA2B, AMPK, PRDM16, β3-adrenergic receptor, CAMK, and SIRT1 have been considered key mediators of energy expenditure and heat production in brown and beige adipocytes (8–12). Animal studies and human studies have found that both cold exposure and exercise can promote browning of adipose tissue (21, 22). However, there are no reports on fruit flies. In this study, we discussed the possibility of improving the expression level of *ucp4c*, *serca*, *octβ3r*, *hamlet*, *ampkα*, *camk II*, and *sirt1* in *Drosophila* and restoring cardiac insufficiency and low exercise capacity caused by HFD through exercise and/or cold exposure. Our main findings are as follows: (1) Under different intervention states, the mRNA expression levels of *ucp4c, serca, octβ3r, hamlet, ampkα, camk II*, and *sirt1* were changed (**Figure 4**). (2) In the NFD group, only exercise can improve the climbing index, which decreased significantly under cold exposure or exercise/cold exposure conditions. In the HFD group, exercise and/or cold exposure significantly increased the climbing index. (3) In the NFD group, exercise can effectively improve cardiac function, including lowering HR, decreasing the risk of AI and FB, and enhancing the pumping capacity. Cold exposure and exercise/cold exposure only partially improved cardiac function. In the HFD group, although all interventions decreased HR and improved FS, cold exposure or exercise/cold exposure increased the risk of arrhythmia and fibrillation to different degrees; only exercise significantly reduced the risk of AI and FL.

**Figure 9:**
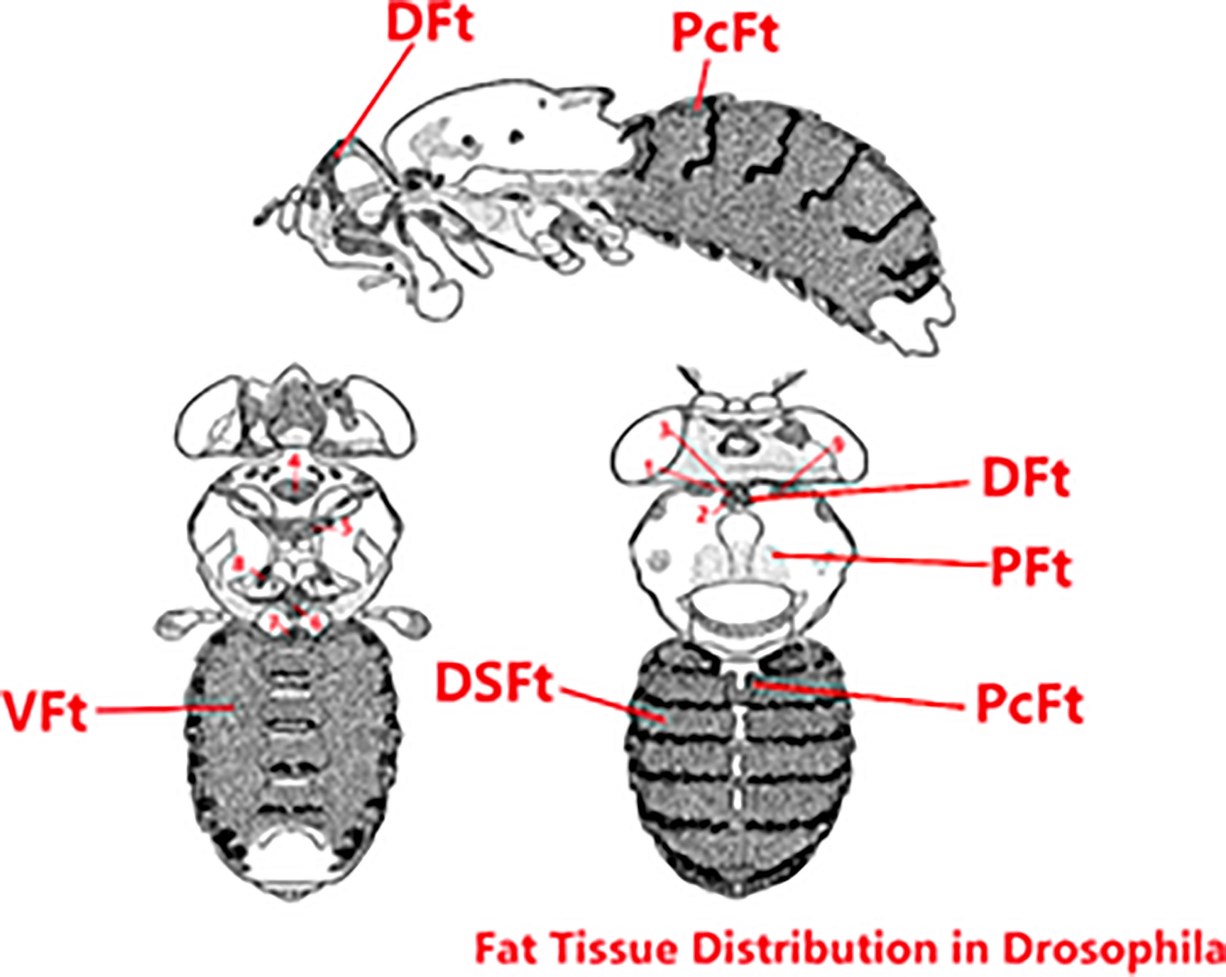
Fat tissue distribution in *Drosophila*. The original image collected from flybase.org was modified. DFt, Deep fat tissue; PFt, Peripheral fat tissue; PcFt, Pericardial fat tissue. VFt, Ventral fat tissue; DSFt, Dorsal fat tissue; 1-9, Breast-specific fat tissue.

In *Drosophila*, as shown in **Figure 9**, fat is distributed throughout the body in six parts: deep fat tissue, peripheral fat tissue, pericardial fat tissue, ventral fat tissue, dorsal fat tissue, and breast-specific fat tissue. We can easily observe the dorsal vessel surrounded by fat, and fat is concentrated around the ventral and dorsal sides; the body structure is relatively simple. *Drosophila*’s relatively short lifespan, its organ systems that perform essentially the same metabolic functions as their vertebrate counterparts, and the sophisticated genetic tools available for studying this species (23) lay a firm foundation for *Drosophila* to be an excellent model for studying obesity, diabetes, cardiomyopathy, and exercise. In addition, the reasons choosing *Drosophila* as the animal model for this research are as follows: (1) Most genes and gene families known to function in metabolic disease and adipose proteins’ structure and function are highly conserved between flies and humans (24). (2) Fruit flies develop obesity, and their associated complications during caloric overload are similar to those of humans (25). (3) The *Drosophila* cardiac system shares many similarities and provides many advantages as a model of cardiovascular diseases (16), the *Drosophila* dorsal vessel is an organ for hemolymph circulation that resembles the vertebrate heart at its transient linear tube stage, and dorsal vessel morphogenesis shares several similarities with early events of vertebrate heart development. In particular, cardiac progenitors are derived from a laterally positioned mesoderm, being specified through the use of conserved cellular induction pathways and downstream transcriptional effectors (14, 15). (4) The *Drosophila* blood system contains cells that are functionally similar to myeloid cells in mammals. As in mammalian systems, multiple sites of hematopoiesis are evident in *Drosophila*, and the mechanisms involved in this process employ many of the same molecular strategies that exemplify blood development in humans (26). (5) *Drosophila* researchers have been able to investigate the impacts of exercise on the animals’ physical fitness, cardiac health, ageing, and more (27). Meanwhile, in fruit flies, previous studies have demonstrated that translational results can be obtained and that can serve to complement mammalian studies (14), and numerous studies have used *Drosophila* as a model for studying exercise (28, 29). Therefore, we decided to explore the expression levels of *ucp4c*, *serca octβ3r*, *hamlet*, *ampkα*, *camk II*, and *sirt1* in *Drosophila* under exercise and/or cold exposure conditions and the effects on HFD-induced cardiac insufficiency and low exercise capacity.

Adipose tissue is categorized into white, brown, and beige. WATs store excess energy as TAGs in one huge single lipid droplet. In total opposition to the specialized role of white adipocytes in energy storage, brown adipocytes have high mitochondrial content and vast numbers of small lipid droplets, and they occupy an important position in uncoupled respiration (30). Particularly, adipose tissue has prominent plasticity properties, and white adipocytes can differentiate into brown-like adipocytes (beige adipocytes) in a process called browning (31). Beige adipose tissue resembles BAT by morphology and function and also has the ability to produce heat.

When the rate of food combustion (decreased metabolic efficiency) or the rate of heat production increases, individual adipocytes receive a signal from the sympathetic nervous system. In brown adipocytes, the hormone and neurotransmitter norepinephrine (NE) activates β3-adrenergic receptors, and then the signal is transmitted *via* cAMP and protein kinase A, causing the release of free fatty acids (FFAs) from TAGs, initiating TAG breakdown. FFAs are involved in the physiological activation of UCP1 and/or the transport mechanism and UCP1 thus allows for mitochondrial combustion of substrates, uncoupled from the production of ATP (10) In beige adipocytes, calcium cycling is promoted by adrenergic activation and Ca^2+^ released from the sarco(endo)plasmic reticulum (SER) is recycled by the SER Ca^2+^-ATPase 2b (SERCA2b) pump (8). Many research studies indicate that Ca^2+^ release also plays a fundamental role in a range of signaling processes in muscles, and RYR1, CASQ, SERCA, and SLN jointly regulate calcium homeostasis (28, 29, 32–35). In *Drosophila*, *ucp4c* is the homologous gene of *UCP1*, *serca* is the homologous gene of *SERCA2B*, *RyR* is the homologous gene of RYR1/RYR2, and no homologous genes of CASQ and SLN were found. In our research, we focus on heart function, and so have little discussion about muscles, which is also a limitation of this study. Therefore, we decided to observe the expression levels of *ucp4c, serca, octβ3, hamlet, ampkα, camk II*, and *sirt1* through the intervention of exercise and/or cold expose to provide a basis for further research.

During exercise, fruit flies need a large amount of energy for muscular activity. Living at low temperatures, fruit flies also need energy to resist the cold environment. As shown in **Figures 2**–**4**, upon loss of fat mass, the fly’s lean size and TAG levels significantly decreased upon energy consumption. TAGs stored in adipose tissue are mobilized to provide fatty acids for energy conversion. These may be the reasons why mRNA expression levels of *ucp4c, serca, octβ3r, hamlet, camk II, ampkα*, and *sirt1* were changed to varying degrees under different intervention states. However, this is not enough to prove whether browning occurs in the flies, and so more experimental studies are needed.

As is well known, obesity is correlated with cardiovascular risk factors. Abdominal obesity is a major risk factor for the development of type 2 diabetes and atherogenic dyslipidemia, which increases the risk of premature coronary heart disease. This increased risk can be largely attributed to high accumulation of abdominal adipose tissue, especially of visceral adipose tissue (36). Studies showed that weight reduction is significantly associated with a lower risk of AF recurrence; a reduction in AF symptoms affects both CVD risk factors and cardiac structure and function (37). So, can the risk of obesity-induced cardiovascular disease be reduced by decreasing abdominal fat levels through exercise and/or cold exposure?

Many studies have verified that regular exercise positively affects the lipid profile and blood pressure, reducing the rate of cardiovascular events and associated mortality (37, 38). This has also been demonstrated in fruit flies by Wen et al. (39). After exercise intervention, a series of molecular changes occur in the vascular system, such as increases in absolute vascular nitric oxide (NO) levels, vascular regeneration, endothelial repair, and cardiac expression of miR-126 (40). All of these changes can effectively prove that exercise significantly helps the prevention of in cardiovascular disease. Our experiment also proves that exercise can restore HFD-induced reductions in cardiac function and strengthen the heart pumping function (**Figures 7**, **8**). In addition, the locomotor ability of fruit flies also significantly improved (**Figure 5**). What about the effect of cold exposure or exercise/cold exposure? Although high lipid consumption relieves abdominal fat accumulation, improves locomotor capacity, slows down the heart rate, and strengthens the heart pumping function, FL significantly increased and AI did not significantly change, which means cold exposure may not be very effective in restoring HFD-induced cardiac insufficiency. Interventions affect cardiovascular responses, which may be altered by underlying cardiovascular disease. Upon cold exposure, HFD fruit flies need to burn fat to fight the cold, which creates a greater pressure load on their heart and may increase the risk of FL and AL. A number of studies have pointed out that cold environments can cause myocardial ischemia, arrhythmia, myocardial hypertrophy, and other cardiac disorders (41, 42). From our findings, we can speculate that, for humans, a cold environment may not be suitable for patients with obesity and cardiovascular disease, which may increase the risk of FL, and that regardless of whether the individuals are healthy or have cardiovascular disease or obesity, exercise seems to be the best approach.

It can be seen from **Figure 5** that cold environments seem to exert no effects on HFD fruit flies’ locomotor capacity. The HFD flies had improved locomotor capacity after losing weight. However, their locomotor capacity was significantly lower in the NFD group. Upon cold exposure, we observed that the fruit flies were more likely to be in a motionless state compared with the control group, in agreement with a previous study which reported that 50% of fruit flies could not maintain flight for 1 s at 15°C (43). We speculate that the decline in locomotor capacity results from a decrease in daily activity, due to which muscular strength is lower than that of normal fruit flies. Kellys (44) indicated that physical inactivity may be particularly deleterious in T2D or in the elderly, and even a short-term reduction in physical activity has significant impact on skeletal muscle protein and carbohydrate metabolism. In addition, lower temperatures can reduce metabolic rates, change muscle contraction activity, and impair muscle physiology (45). Moreover, the CM Williams’ research has shown that low temperatures induce a reversible loss of neuromuscular coordination in insects (46). In conclusion, compared with cold exposure or exercise/cold exposure conditions, only regular exercise can truly help restore HFD-induced cardiac insufficiency and low exercise capacity.

## Methods

### Fly Stocks and Culture

We used wild-type *Drosophila melanogaster* strain w1118 virgin female flies (collected within 8 h after eclosion). NFD fruit flies were housed in an incubator at 25°C while HFD flies were housed in an incubator at 22–24°C and 50% relative humidity under a 12/12-h light/dark cycle. Fresh food was provided once every 2 days without anesthesia. High-fat medium was made by mixing 30% coconut oil and 70% standard medium.

### Exercise Training Device and Protocols

Flies were randomly divided into eight groups: the NFD control group (NFD-C), the NFD exercise group (NFD-E), the NFD cold exposure group (NFD-CA), the NFD exercise/cold exposure group (NFD-EC), the HFD control group (HFD-C), the HFD exercise group (HFD-E), the HFD cold exposure group (HFD-CA), and the HFD exercise/cold exposure group (HFD-EC). According their natural negative geotaxis behavior, we designed an exercise device (19). Vials were horizontally loaded (with feed and housing 20 flies each) into a steel tube that was rotated about its horizontal axis at a gear control shafting speed. To stimulate most flies to climb, each vial was rotated along its long axis. Few flies that failed to climb were actively walking on the inner wall of the vial. Vials rotated at 0.16 rev/s, and flies exercised for 2.5 hours per day.

### Cold Exposure

For *D. melanogaster*, 18–25°C is an acclimation temperature. When the temperature falls below 10°C, the development of *D. melanogaster* will halt (34). Fat content significantly reduces when temperatures fall below 13°C. Therefore, at 1–7 days after emergence, vials were kept at 15°C for 2.5 hours.

### qRT-PCR

**Figure 10:**
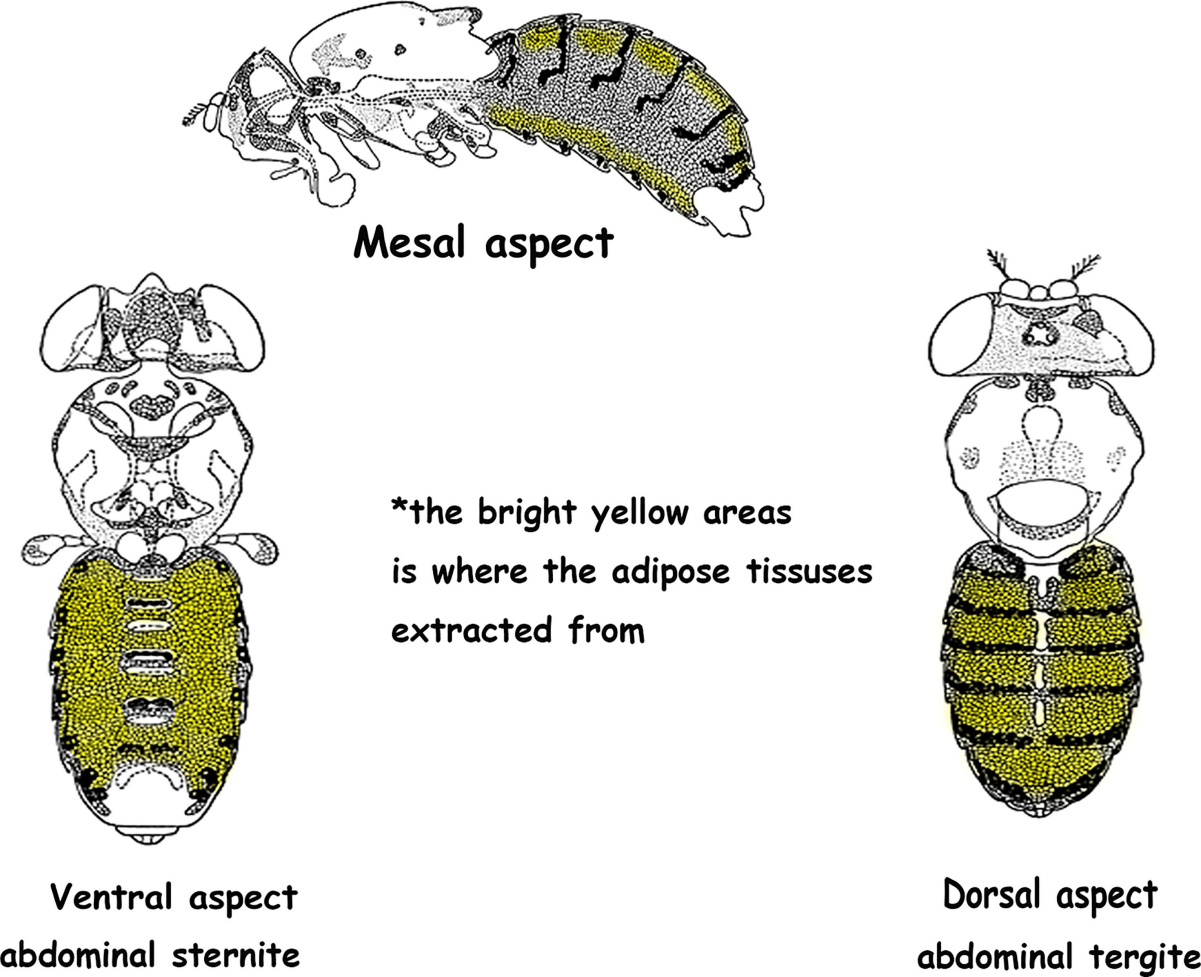
Adipose tissue was extracted from the bright yellow areas. *The original image was collected from flybase.org and modified.

Ten abdominal subcutaneous adipose tissue samples were collected from each group and total RNA was isolated from tissue using TRIzol (Invitrogen, CA) according to the manufacturer’s protocol. Adipose tissues were extracted from the abdominal sternite and the abdominal tergite (**Figure 10**). cDNA was generated using Superscript III reverse transcriptase (Invitrogen, CA) and used as the templates for quantitative real-time PCR. Real-time PCR was performed using SYBR green using an ABI7300 Real-time PCR Instrument (Applied Biosystems). The tested genes’ relative abundance was calculated by the 2^−ΔΔCt^ method. Primers used for expression analysis were as follows:

Primer sequences of *ucp4c*: F: 5′-CGGCTTCTCGGCAATGGTGAC-3′; R: 5′-CTTCCTCGTTCCGCTCGTTCTG-3′.

Primer sequences of *serca*: F: 5′-TGCTGGTGAAGATTCTGCTGTTGG-3′; R: 5′-CACGGCGTTGGCTATCAGGATAAG-3′.

Primer sequences of *Octβ3R*: F: 5′- TGTGGTCAACAAGGCCTACG-3′; R: 5′- GTGTTCGGCGCTGTTAAGGA-3′.

Primer sequences of *hamlet*: F: 5′- ATAGATCCTTTGGCCAGCAGAC-3′; R: 5′- AGTACTCCTCCCTTTCGGCAAT-3′.

Primer sequences of *AMPKα*: F: 5′- GGCCATCGCTTACCATCTGA -3′; R: 5′- ATCGATGATTGGGGAACGGG-3′.

Primer sequences of *Camk II*: F: 5′- ACAACCATTTTAAATCCTCATGTGC-3′; R: 5′- TGTGCATGTCCTTGTTATCA-3′.

Primer sequences of *Sirt1*: F: 5′- TAGAGCCTCACATGCAAGCTCTA-3′; R: 5′- GCCAATCATAAGATGTTGCTGAAC-3′.

TAG levels were measured by ELISA (TAG ELISA Kits, MLBIO) as follows: (1) Samples (*n* = 15, three groups) were homogenized with grinders and centrifuged for 15 min (4000 rpm, 4°C). (2) Standard wells were set and 50 μl standard solution was added to the standard wells. (3) To the sample wells, 40 μl sample solution and 10 μl of the sample were added and mixed gently. (4) Except for the blank wells, 100 μl of HRP-conjugate reagent II was added to each well. (5) Plates were incubated for 1 h at 37°C. (6) After centrifugation, wells were washed five times with washing buffer for 30 s. (7) Stop solution (50 μl) was added to each well to stop the reactions. (8) The blank values were subtracted from all others.

### Oil Red O Staining

ORO staining was used to quantify lipids. (1) Fruit flies were dissected and the adipose tissue was exposed in phosphate-buffered saline (PBS). (2) PBS was removed and samples were fixed with 4% paraformaldehyde for 20 min. (3) Samples were washed three times with PBS for 10 min. (4) Oil Red O reagent was dropped on the samples, which were incubated for 1 h at room temperature. (5) The samples were washed three times with PBS for 10 min. (6) The samples were transferred to a slide with mounting solution. (7) Photos were taken with a Leica stereo microscope.

### Semi-Intact *Drosophila* Heart Preparation and Heartbeat Analysis

First, 30 flies were fixed back down on a Petri dish after applying anesthesia with FlyNap for 2–3 min. Second, the head was rapidly removed, artificial hemolymph was added (visualizing the beating heart in *Drosophila*), and then all internal organs except the heart and any abdominal fat were removed. Oxygen was pumped for 15 min at room temperature. Fruit flies’ heartbeats were recorded by an EM-CCD high-speed camera (video at 130 fps for 30 s), and the cardiogram data were recorded by HC Image software. Semi-Automated Optical Heartbeat Analysis (SOHA, kindly gifted by Ocorr and Bodmer), a software program that accurately quantifies heart rate (HR), heart period (HP), diastolic diameter (DD), systolic diameter (SD), systolic interval (SI), diastolic interval (DI), arrhythmicity index (AI), fibrillations (FL), and the percentage of fractional shortening (FS), was used to assess functional cardiac parameters of fruit flies.

### Negative Geotaxis Assay

Flies from each group were transformed into the climbing apparatus, which consisted of an 18-cm-long glass tube with an inner diameter of 2.8 cm (sponges were placed in the ends of the tube to prevent escape yet allow air exchange), 20 pieces per tube, 5 tubes with in total 20 fruit flies per tube, 5 tubes in total (47). Fruit flies were gently shaken to the bottom and climbed up the wall of the tube because of the negative geotaxis instinct. The first three times were considered adaptive climbing, and the fourth, fifth, and sixth climbing heights reached by the fruit flies at the end of 5 seconds were recorded by a video camera. The climbing index is the average climbing height of the flies in each vial.

### Statistical Analysis

SPSS version 25.0 software was used to analyze all data. Data are presented as the mean ± standard error of the mean (SEM). The statistical techniques performed to identify differences between the groups were multi-way ANOVA and least significant difference (LSD) tests. Statistical significance was set at *P* < 0.05.

## Conclusion

The mRNA expression levels of *ucp4c, serca, octβ3r, hamlet, ampkα, camk II*, and *sirt1* were changed to varying degrees under different intervention states (exercise and/or cold exposure), which laid the foundation for future research. Compared with cold exposure or exercise/cold exposure, exercise is the best way to restore HFD-induced cardiac dysfunction, which facilitates the study of the mechanisms underlying brown and beige adipose tissue activity and generation in obesity and other metabolic diseases.

## Data Availability Statement

The original contributions presented in the study are included in the article/supplementary material. Further inquiries can be directed to the corresponding author.

## Author Contributions

TH and LZ conceived and designed the experiments and wrote the manuscript. TH, XJ, JL, MD, SZ, TW and QL collected the samples. TH, XJ, JL, JFYL, and GZ performed the experiments. TH, XJ, JL, analyzed the data. All authors have read and approved the final manuscript.

## Funding

This work was supported by the National Natural Science Foundation of China (No. 31671243 and 32071175), the Hunan Province Graduate Education Innovation Project and Professional Ability Enhancement Project Fund (Project Number: CX20200533) and Key scientific research project of Hunan Education Department (Project Number: 19A328).

## Conflict of Interest

The authors declare that the research was conducted in the absence of any commercial or financial relationships that could be construed as a potential conflict of interest.

## Publisher’s Note

All claims expressed in this article are solely those of the authors and do not necessarily represent those of their affiliated organizations, or those of the publisher, the editors and the reviewers. Any product that may be evaluated in this article, or claim that may be made by its manufacturer, is not guaranteed or endorsed by the publisher.
