# Corrigendum: Exercise and/or Cold Exposure Alters the Gene Expression Profile in the Fat Body and Changes the Heart Function in *Drosophila*

**DOI:** 10.3389/fendo.2022.920358

**Published:** 2022-07-07

**Authors:** Ting Huang, Xiaoyi Jian, Jinglin Liu, Lan Zheng, Fang Qiu Li, Ding Meng, Tongquan Wang, Shihu Zhang, Yi Liu, Zhilong Guan, Jiadong Feng

**Affiliations:** Key Laboratory of Physical Fitness and Exercise Rehabilitation of Hunan Province, Hunan Normal University, Changsha, China

**Keywords:** exercise, cold expose, cardia function, ucp4c, SIRT1

In the original article, there was a mistake in **Figure 4** as originally published. **Figure 4** was incomplete and omitted a set of results. The corrected **Figure 4** appears here.

**Figure 4:**
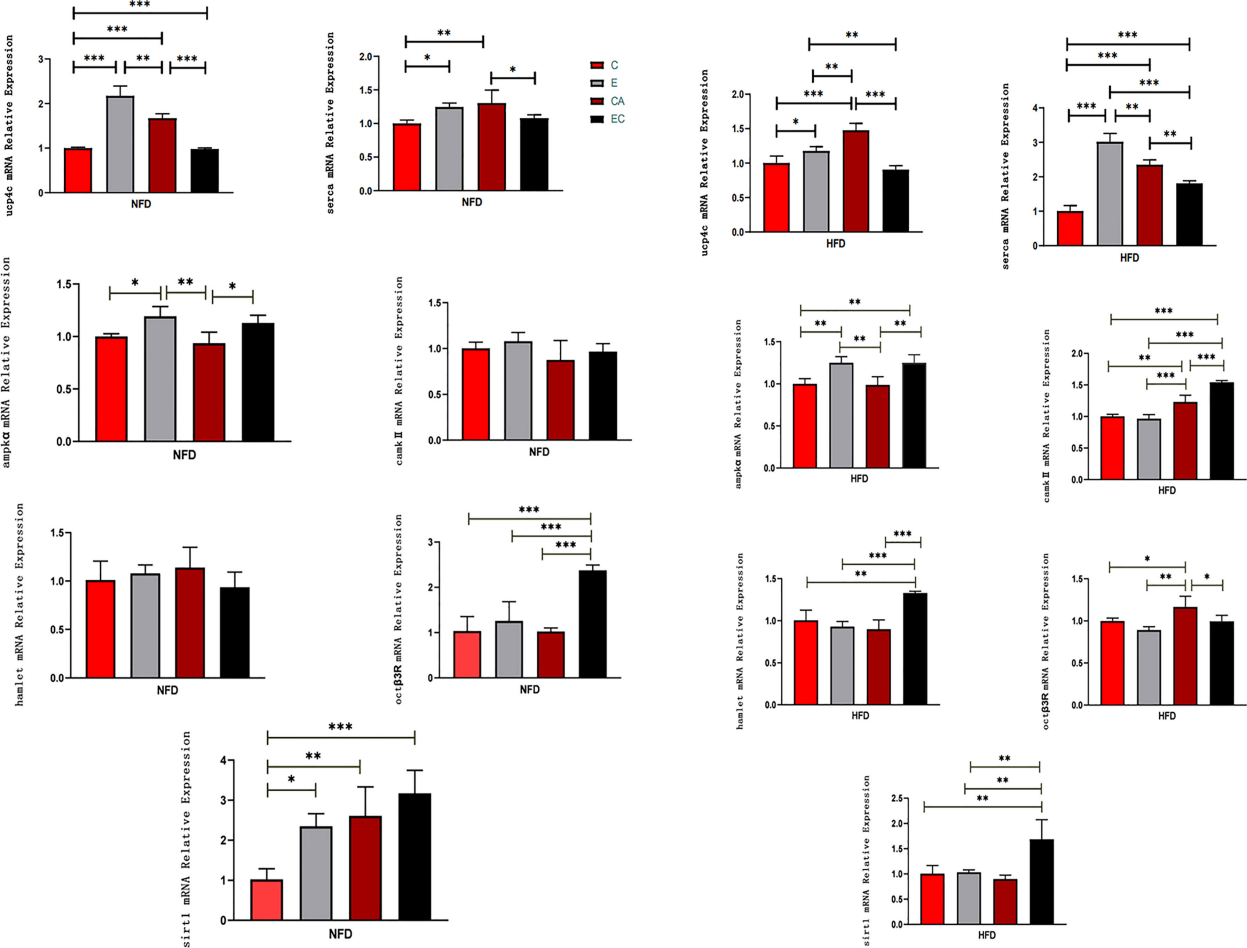
mRNA expression levels of *ucp4c, serca, octβ3r, hamlet, ampkα, camk II*, and *sirt1* under different interventions. *P < 0.05, **P < 0.01, ***P < 0.001.

**Figure 4** **caption** mRNA expression levels of *ucp4c*, *serca*, *octβ3r*, *hamlet*, *ampkα*, *camk II*, and *sirt1* under different interventions. *P < 0.05, **P < 0.01, ***P < 0.001.

In the *Results*, subsection *Exercise and/or Cold Exposure Reduced the Amount of Fat in Fruit Flies* as originally published, the following sentence contained the wrong table citation: “According to the multi-factor analysis of variance (ANOVA) of triacylglycerol (TAG) levels (**Table 3**), feeding patterns and interventions exert major effects on TAG levels and have no interaction.” The correct citation is: “According to the multi-factor analysis of variance (ANOVA) of triacylglycerol (TAG) levels (**Table 1**), feeding patterns and interventions exert major effects on TAG levels and have no interaction.”

**Table 1:** Multi-factor analysis of variance of TAG levels.

Parameters ofcardiac function	Source	Type III sum of squares	df	Mean square	F	Sig.
Heart rate	Feeding patterns	1.078	1	1.078	5.164	0.025
	Intervention methods	6.485	3	2.162	10.357	0.000
	Feeding patterns × intervention methods	2.570	3	0.857	4.104	0.008
Heart period	Feeding patterns	0.023	1	0.023	0.502	0.480
	Intervention methods	1.282	3	0.427	9.135	0.000
	Feeding patterns × intervention methods	0.492	3	0.164	3.505	0.017
Diastolic intervals	Feeding patterns	0.132	1	0.132	3.625	0.059
	Intervention methods	0.597	3	0.199	5.470	0.001
	Feeding patterns × intervention methods	0.378	3	0.126	3.463	0.018
Systolic intervals	Feeding patterns	0.011	1	0.011	4.966	0.027
	Intervention methods	0.059	3	0.020	9.074	0.000
	Feeding patterns × intervention methods	0.004	3	0.001	0.664	0.576
Arrhythmia	Feeding patterns	0.270	1	0.270	120.211	0.000
	Intervention methods	0.120	3	0.040	17.756	0.000
	Feeding patterns × intervention methods	0.028	3	0.009	4.110	0.008
Diastolic diameter	Feeding patterns	7186.813	1	7186.813	2.862	0.093
	Intervention methods	77704.678	3	25901.559	10.313	0.000
	Feeding patterns × intervention methods	29669.679	3	9889.893	3.938	0.010
Systolic diameter	Feeding patterns	23478.422	1	23478.422	13.795	0.000
	Intervention methods	17265.249	3	5755.083	3.382	0.020
	Feeding patterns × intervention methods	24360.895	3	8120.298	4.771	0.003
Fractional shortening	Feeding patterns	0.936	1	0.936	198.541	0.000
	Intervention methods	0.118	3	0.039	8.313	0.000
	Feeding patterns × intervention methods	0.017	3	0.006	1.203	0.311
Fibrillations	Feeding patterns	1.247	1	1.247	13.584	0.000
	Intervention methods	3.159	3	1.053	11.475	0.000
	Feeding patterns × intervention methods	0.643	3	0.214	2.335	0.076

**Table 3:** Multi-factor analysis of variance of locomotor capacity.

Source	Type III sum of squares	df	Mean square	F	Sig.
Feeding patterns	75692.644	1	75692.644	104.789	0.000
Intervention methods	36609.452	3	12203.151	16.894	0.001
Feeding patterns × intervention methods	1608.249	3	536.083	0.742	0.556

In the *Discussion* (seventh paragraph) as originally published, the following sentence contained the wrong figure citation: “In addition, the locomotor ability of fruit flies also significantly improved (**Figure 6**)” The correct citation is: “In addition, the locomotor ability of fruit flies also significantly improved (**Figure 5**).”

**Figure 5:**
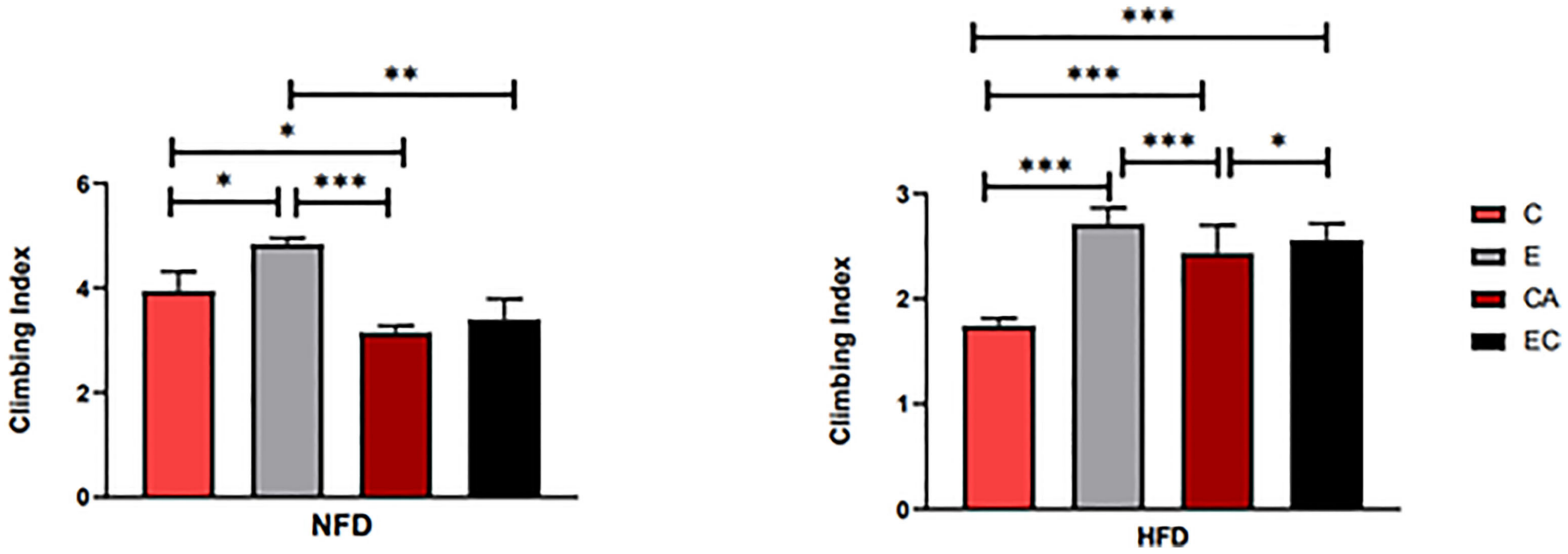
Flies’ locomotor ability under different interventions. *P < 0.05, **P < 0.01, ***P < 0.001.

**Figure 6:**
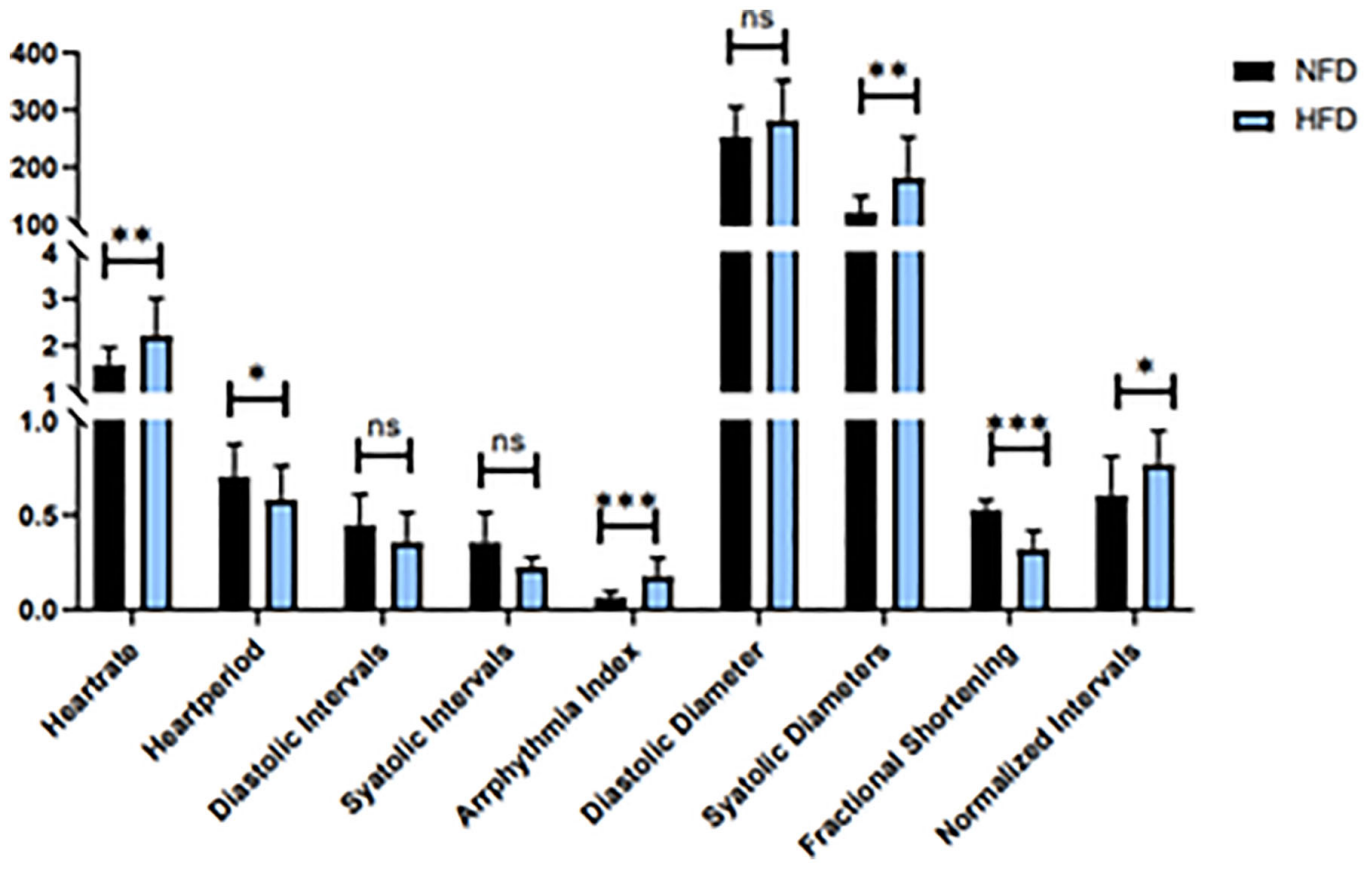
Heart function in HFD and NFD fruit flies. *P < 0.05, **P < 0.01, ***P < 0.001, ns P > 0.05.

In the *Discussion* (eighth paragraph) as originally published, the following sentence contained the wrong figure citation: “It can be seen from **Figure 7** that cold environments seem to exert no effects on HFD fruit flies’ locomotor capacity. The HFD flies had improved locomotor capacity after losing weight.” The correct citation is: “It can be seen from **Figure 5** that cold environments seem to exert no effects on HFD fruit flies’ locomotor capacity. The HFD flies had improved locomotor capacity after losing weight.”

**Figure 7:**
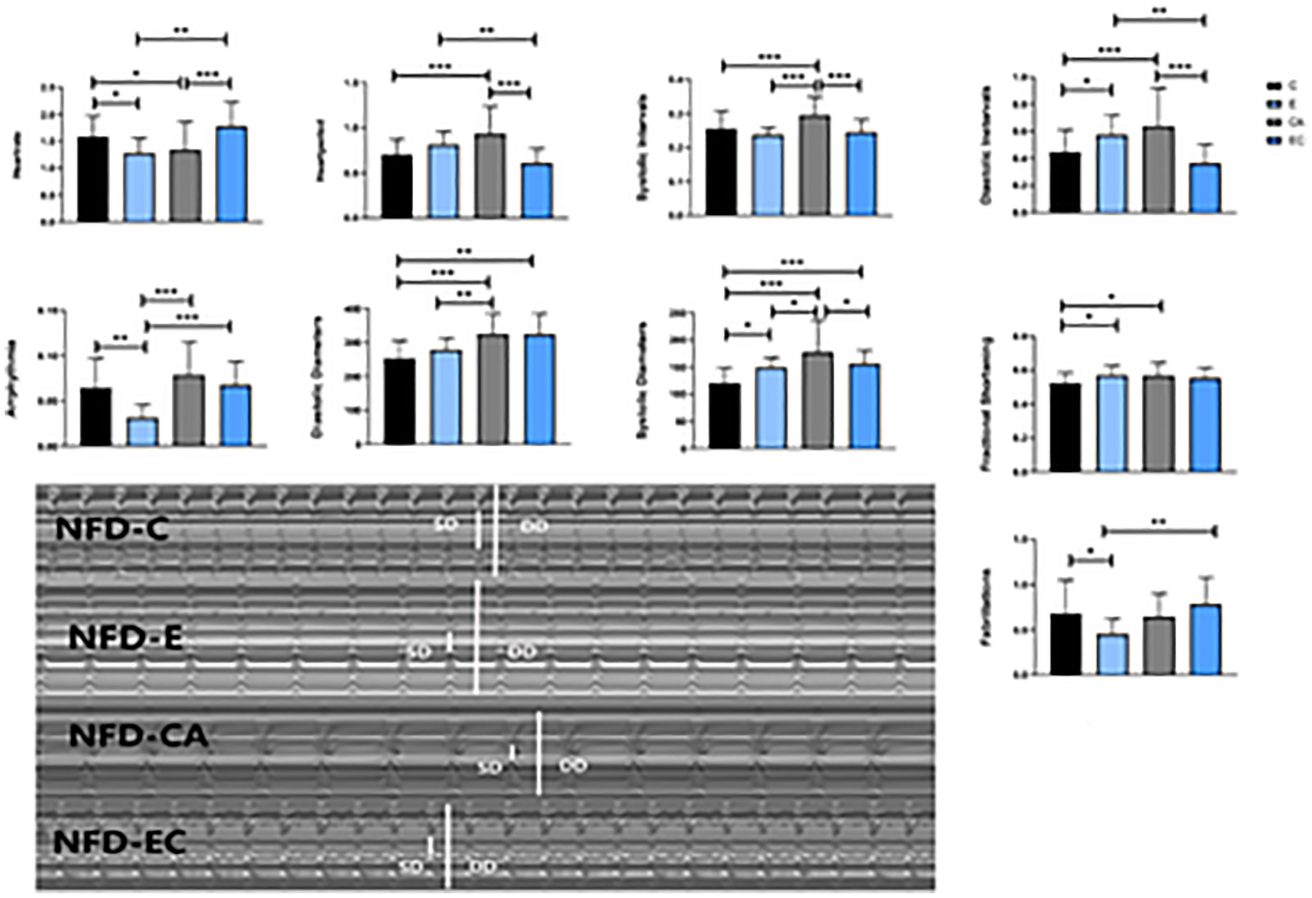
Changes in heart function of NFD fruit flies. *P < 0.05, **P < 0.01, ***P < 0.001.

In the *Methods*, subsection *qRT-PCR* as originally published, the following sentence incorrectly stated that the expression-analysis primers were shown in **Table 3**: “**Table 3** shows the primers used for expression analysis”. The sentence should read “Primers used for expression analysis were as follows:”.

The authors apologize for these errors and state that they do not change the scientific conclusions of the article in any way. The original article has been updated.

## Publisher’s Note

All claims expressed in this article are solely those of the authors and do not necessarily represent those of their affiliated organizations, or those of the publisher, the editors and the reviewers. Any product that may be evaluated in this article, or claim that may be made by its manufacturer, is not guaranteed or endorsed by the publisher.

